# Impaired mentalizing in depression and the effects of borderline personality disorder on this relationship

**DOI:** 10.1186/s40479-021-00153-x

**Published:** 2021-05-04

**Authors:** R. P. Rifkin-Zybutz, P. Moran, T. Nolte, Janet Feigenbaum, Brooks King-Casas, London Personality and Mood Disorder Consortium, P. Fonagy, R. P. Montague

**Affiliations:** 1 Centre for Academic Mental Health, University of Bristol, Oakfield House, Bristol, BS8 2BN UK; 2 Virginia Tech Carilion Research Institute (BK-C, PHC), Roanoke, USA; 3 Department of Psychology (BK-C, PHC), Virginia Tech, Roanoke, USA; 4 Department of Psychiatry (BK-C, PHC), Virginia Tech Carilion School of Medicine, Roanoke, USA; 5 Wellcome Department of Imaging Neuroscience, University College London, London, UK; 6 Anna Freud National Centre for Children and Families, University College London, London, UK; 7 Research Department of Clinical, Educational, and Health Psychology, University College London, London, UK

**Keywords:** Mentalization, BPD, Depression

## Abstract

**Background:**

Mentalizing, the ability to understand the self and others as well as behaviour in terms of intentional mental states, is impaired in Borderline Personality Disorder (BPD). Evidence for mentalizing deficits in other mental disorders, such as depression, is less robust and these links have never been explored while accounting for the effects of BPD on mentalizing. Additionally, it is unknown whether BPD symptoms might moderate any relationship between depressive symptoms and mentalizing.

**Methods:**

Using multivariate regression modelling on cross-sectional data obtained from a sample of 274 participants recruited from clinical settings, we investigated the association between mentalizing impairment and depression and examined whether this was moderated by the presence and number of concurrent BPD symptoms, while adjusting for socio-demographic confounders.

**Results:**

Impaired mentalizing was associated with depressive symptoms, after adjustment for socio-demographic confounders and BPD symptoms (*p* = 0.002, β = − 0.18). BPD symptoms significantly moderated the association between impaired mentalizing and depressive symptoms (*p* = 0.003), with more severe borderline symptoms associated with a stronger effect of poor mentalization on increased depressive symptoms.

**Conclusion:**

Mentalizing impairments occur in depression even after adjusting for the effect of BPD symptoms. Our findings help further characterise mentalizing impairments in depression, as well as the moderating effect of BPD symptoms on this association.. Further longitudinal work is required to investigate the direction of association.

**Supplementary Information:**

The online version contains supplementary material available at 10.1186/s40479-021-00153-x.

## Introduction

Mentalizing refers to the ability to understand behaviours and actions in oneself and others in terms of internal mental states. Skilful mentalization is accurate, coherent, rich in depth and flexible [1] and is thought to develop best within stable and secure relationships within childhood [2]. Therefore, we might expect mentalization to be compromised across a broad range of psychiatric disorders reflecting impaired development in the face of stress [3]. Indeed, Mentalization has been found to be impaired in individuals with borderline personality disorder (BPD) [4, 5]. Indeed, it has been proposed that mentalizing deficits may underlie key features of BPD [6]. For example, elevated sensitivity to interpersonal rejection, a core feature of BPD, could be considered a failure of adequately mentalizing the intentions of others [7]. Inadequate mentalization may therefore underlie excessive responses such as hostile behaviours in response to rejection, due to a misreading of both intent and threat [8]. Individuals with BPD are particularly prone to pre-mentalistic states, such as psychic equivalence (regarding negative thoughts as indicating or being equivalent to a negative reality) when they experience strong negative affect [9, 10]. These deficits in mentalizing are thought to arise from disordered attachment and negative experiences of social communication [11].

The occurrence of mentalizing deficits in major depressive disorder (MDD) has been hypothesized [12] and previously reported, although the evidence is less clear cut than in the case of BPD [9]. While two studies reported mentalizing deficits in psychiatric inpatients with depression compared to healthy controls [13, 14], another found no difference in reflective functioning, an operatilization of mentalizing, between chronically depressed patients receiving psychotherapy and healthy controls [15]. Poor mentalizing could precipitate low mood by increasing sensitivity to interpersonal rejection and failure to calibrate negative self-representation through the view others may hold about oneself [11].

Major Depressive disorder (MDD) and BPD often occur concurrently [16] and it is estimated that over 80% of individuals with BPD experience an episode of MDD over the course of their life [17]. From a clinical perspective, the occurrence of this co-morbidity is important because BPD co-morbidity has been shown to reduce the chance of MDD remitting [16] and is more common in chronic depression.

Impairments in mentalizing therefore appear to be a transdiagnostic construct that may contribute to deficits across both MDD and BPD. Poor mentalisation may be particularly harmful in BPD where mentalizing deficits interface with pre-existing negative self-representation. This increases vulnerability to pre-mentalizing modes, such as psychic equivalence that may uniquely contribute to the onset of depressive symptoms [10].

In this study, we therefore set out to investigate whether there is an association between impairment in mentalizing and depressive symptoms and furthermore, to test whether borderline symptoms moderated this relationship, hypothesizing that the links between poor mentalisation and depression should be stronger in those with higher levels of BPD symptoms.

## Methods

### Recruitment

Ethics approval was gained for the collection and use of all participants’ data (Research Ethics Committee Wales, 12/WA/0283).

Potential participants were aged 18–60 and consisted of three samples all recruited from the London area: i) a sample of 83 patients with BPD, recruited from specialist clinical services for people with BPD and confirmed via structured clinical interview (SCID II); ii) a sample of 119 patients with affective disorder, recruited from local NHS psychological treatment services; iii) a sample of 72 healthy adult control participants recruited from a range of community settings (online, notice boards, universities, and job centres). In terms of eligibility criteria, all participants were required to be over the age of 18 years and fluent in English. Individuals were excluded if they were psychotic, had a learning disability, or if they had a neurological disorder.

All participants were assessed at the offices of University College London. Participants were remunerated for their time and expenses incurred.

Table 1 presents the sample’s demographics.

**Table 1:** Baseline Characteristics of Sample

Socio-demographic variables			
Gender	Male	71/274	(26%)
	Female	200/274	(73%)
	Other	3/274	(1%)
	Missing	0/274	(0%)
Ethnicity	White	175/274	(64%)
	Black	31/274	(11%)
	Mixed	26/274	(10%)
	Asian	33/274	(12%)
	Other	8/274	(3%)
	Missing	1/274	(0%)
Age (years): mean (SD)	Missing = 0/274 (0%)	30.3	(10.2)
Years in Education: mean (SD)	Missing = 28/274 (10%)	14.6	(3.0)
Household Income	Less than £20,000	113/274	(42%)
	£20,000 – £35,000	62/274	(23%)
	More Than £35,000	96/274	(35%)
	Missing	3/274	(1%)
Employment Type	Employed	132/274	(48%)
	Student/Apprenticeship	58/274	(21%)
	Retired/Carer	8/274	(3%)
	Unemployed	75/274	(27%)
	Missing	1/274	(0%)
Questionnaire Baseline Measures			
PAIS-BOR: mean (SD)		38.8	(16.5)
PAIS-BOR Category	Not case (≤38)	129/274	(47%)
	Traits (> 38)	113/274	(41%)
	Features (> 60)	32/274	(12%)
MZQ scores: mean (SD)		25.9	(12.1)
BDI II scores: mean (SD)		26.4	(16.5)
Clinical category	Minimal Depression (< 14)	74/274	(27%)
	Mild Depression (14–19)	28/274	(10%)
	Moderate Depression (20–28)	47/274	(17%)
	Severe Depression (> 29)	125/274	(46%)

## Questionnaires

Mentalizing was assessed with the Mentalisation Questionnaire (MZQ) a 15-item, previously validated self-report questionnaire [5]. Scores on each item range from 0 to 4 on each item with lower scores indicating worse mentalizing, with a possible range of scores from 0 to 60.

Depression was assessed with the Becks Depression Inventory -II (BDI-II) [18], a well validated and widely used measure of depression. The BDI-II consists of 21 questions on which participants are scored from 0 to 3 for a maximum of 63. Standard clinical groupings used from this are; minimal depression (BDI-II < 14), mild depression (BDI-II 14–19), moderate depression (BDI-II 20–28) and severe depression (BDI-II > 29).

Borderline personality traits were assessed with the Personality Assessment Inventory – Borderline sub-section (PAI-BOR) [19]. This has been validated across a wide range of ages and genders [20]. A total PAI-BOR raw score of greater than 38 indicates significant BPD features, while a score of 60 or more indicates levels of functioning typically associated with a diagnosis of BPD.

### Statistical analysis

Software package STATA v15 was used for statistical analysis [21].

Simple descriptive statistics were used to characterise the sample. Visual examination of scatter plots and residual plots were undertaken to check linear regression assumptions. Linear regression models were used to model the association between mentalizing impairment and depressive symptoms. Simple linear regression, without adjustment, was used to obtain univariate associations between mentalizing, depressive symptoms and BPD symptoms. The association between depressive symptoms and mentalizing was then assessed in a fully adjusted model accounting for age, gender, household income, ethnicity, years in education, current employment, and BPD symptoms. To test whether BPD symptoms moderated the association between mentalizing and depression, we fitted an interaction term between BPD and MZQ score and used a likelihood ratio test to assess the statistical significance of the interaction term.

## Results

### Baseline characteristics

Two hundred and 74 participants had complete data for age, gender, BDI-II, MZQ and PAIS scoring, 245 of whom also had complete data for socio-demographic characteristics. The total pooled sample was predominantly female (73%), poor (42% - household income <£20,000) and white (64%) with a mean age of 30.3 (SD = 10.8) years. The mean PAI-BOR score in the pooled sample was 38.8 (SD = 16.5) and mean BDI-II score was 25.9 (SD = 12.1) (Table 1).

### Univariate associations

Worsening mentalizing (lower scores) was strongly associated with an increase in the severity of depressive symptoms (β = − 0.63 (− 0.53 to − 0.72), *p* < 0.001) as well as with BPD symptoms (β = − 0.71 (− 0.62 to − 0.78), p < 0.001). The number of BPD symptoms was also strongly associated with the severity of depressive symptoms (β = 0.76 (0.68 to 0.84), *p* < 0.001).

### Multivariate associations

Following adjustment for age, gender, household income, ethnicity, years in education, current employment and the number of BPD symptoms, impairment in mentalizing remained significantly associated with the severity of depressive symptoms but the association was substantially attenuated (β = − 0.18 (− 0.07 to − 0.29), *p* = 0.002). **(**Table 2**– found at end of file).**

**Table 2:** Linear regression model displaying the association between mentalisation and depressive symptoms, adjusted for socio-demographic factors and co-morbid BPD symptoms

Regression On Depressive Symptoms	Coefficient (95% Ci)	Β – standardised coefficient (95% Ci)	*P* Value
Mentalisation – MZQ (Per unit increase – improvement in mentalizing)	**−0.25 (− 0.09 to − 0.40)**	**−0.18 (− 0.07 to − 0.29)**	**0.002**
Borderline Symptoms – PAI-BOR (Per unit increase – more borderline symptoms)	**0.62 (0.50 to 0.73)**	**0.62 (0.50 to 0.74)**	**< 0.001**
Age (Per Year)	**0.03 (−0.11 to 0.18)**	**0.02 (−0.07 to 0.11)**	**0.65**
Years In Education (Per Year)	**0.13 (−0.33 to 0.58)**	**0.02 (−0.06 to 0.11)**	**0.58**
Ethnicity	**Test for overall effect:** ***p*** **= 0.95**		
White	**Baseline**		
Black	**0.07 (−4.38 to 4.53)**	**0.00 (−0.27 to 0.27)**	**0.98**
Mixed	**0.17 (−4.39 to 4.73)**	**0.01 (−0.27 to 0.29)**	**0.94**
Asian	**−0.02 (−4.52 to 4.47)**	**0.00 (− 0.27 to 0.27)**	**0.99**
Other	**−3.07 (−10.67 to 4.53)**	**−0.19 (− 0.65 to 0.27)**	**0.43**
Gender	**Test for overall effect:** ***p*** **= 0.25**		
Male	**Baseline**		
Female	**2.61 (−0.58 to 5.79)**	**0.16 (−0.04 to 0.35)**	**0.11**
Other	**2.68 (−9.82 to 15.18)**	**0.16 (−0.60 to 0.92)**	**0.67**
Household Income	**Test for overall effect:** ***p*** **= 0.67**		
Less Than £20,000	**Baseline**		
£20,000 To £35,000	**0.54 (−3.20 to 4.29)**	**0.03 (−0.19 to 0.26)**	**0.78**
More Than £35,000	**1.48 (−1.91 to 4.86)**	**0.09 (−0.12 to 0.30)**	**0.39**
Employment	**Test for overall effect:** ***p*** **= 0.08**		
Employed	**Baseline**		
Student/Apprentice	**1.80 (−1.99 to 5.60)**	**0.11 (−0.12 to 0.34)**	**0.35**
Retired/Carer	**1.81 (−6.15 to 9.76)**	**0.11 (−0.37 to 0.59)**	**0.66**
Unemployed	**4.55 (0.99 to 8.10)**	**0.28 (0.06 to 0.49)**	**0.01**
Constant	**1.12**		
Number of Observations	**245**		
Adjusted R^2^	**0.61**		

### Effect modification of the association between mentalization and depression, by number of BPD symptoms

Likelihood ratio testing revealed that there was a significant interaction between mentalizing impairment and BPD symptoms in the model of depressive symptoms (χ^2^ = 8.79, *p* = 0.003). Higher levels of BPD symptoms increased the strength of the association between mentalisation and depressive symptoms (see Supplementary Table 1 for output from the multivariable model including the interaction term).

**Fig. 1:**
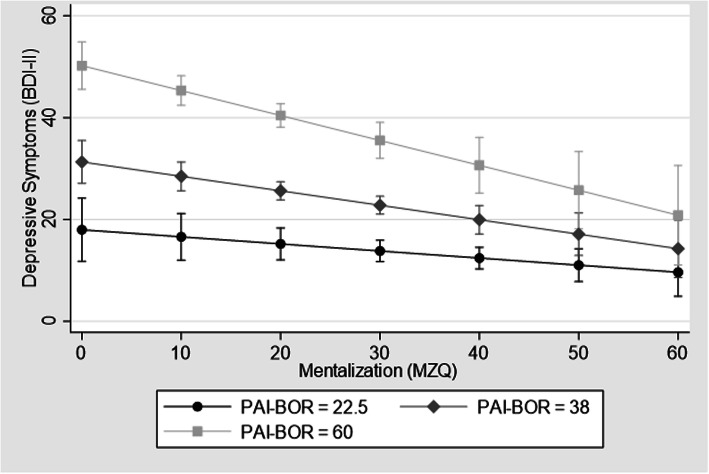
The association between mentalization and depression, stratified by levels of Borderline Personality disorder symptoms. The association between mentalizing and depressive symptoms at 3 key cut-points on the PAIS-BOR. Worsening mentalization is more strongly associated with higher depression scores at higher levels of borderline symptomology

Figure 1 displays the association between mentalizing and depressive symptoms at 3 key cut-points on the PAIS-BOR: 22.5 (the mean PAIS-BOR score in healthy controls in this sample) as well as at pre-specified cut points of 38 (subsyndromal borderline traits) and 60 (features of borderline personality disorder). (Fig. 1).

## Discussion

In this cross-sectional clinical sample, impairment in mentalizing was significantly associated with depressive symptoms. The association was maintained after adjusting for the effects of socio-demographic confounders as well as adjustment for the presence of BPD symptoms, although adjusting for the latter attenuated the strength of the association. This finding is consistent with previous studies suggesting the existence of an independent link between depression and impairment in mentalizing but suggests that a large proportion of the unadjusted relationship is accounted for by covariance with BPD symptoms [6, 9, 13, 14]. To our knowledge, no prior study of this link has accounted for the potential effects of BPD [13–15]. It is also worth acknowledging that depression is a heterogenous entity and it may be that mentalizing deficits contribute to the aetiology of certain subtypes of depression more than others. For example, mentalization deficits may play a more substantial role in the development of anaclitic depression among individuals prone to rejection sensitivity, as opposed to the development of introjective depression among those more prone to self-criticism [8]. This potentially warrants exploring in future studies.

Furthermore, we found that the association between depression and mentalisation was moderated by the severity of BPD symptoms; the association between poorer mentalizing and depression was strongest among those with high levels of BPD symptoms.

Given the cross-sectional nature of this study, the findings need to be interpreted cautiously. While our observed findings help support the psychological theory that mentalization deficits contribute to vulnerability to depression among people with BPD, it is plausible that depression reduces the ability to mentalize, as part of a wider range of effects on mental state [13]. These effects may be more pronounced in those with BPD who have pre-existing mentalisation deficits. Another possible explanation is that impairment in mentalization acts as an independent vulnerability factor for depression, with this effect more pronounced in individuals with co-morbid BPD symptoms.

Our findings require replication and further examination in a longitudinal study to establish the direction of association. If mentalization deficits precede the onset of depression, this raises the prospect of mentalization-based treatment (MBT) [22] – an evidence-based treatment for BPD – being applied as a potential prevention strategy for depression.

Our study has several strengths. The size of our sample is considerably larger than previous studies in the field [13, 14] allowing us to examine interaction effects with greater precision. Furthermore, our use of symptom measures, as opposed to categorically defined diagnostic categories allowed us to investigate the links between depressive symptoms and mentalizing with adequate statistical power. Patient samples were recruited from busy NHS settings adding to the generalisability of findings. The findings however need to be considered in the context of certain methodological limitations. The cross-sectional nature of the data means limits our ability to make inferences about temporal sequence underlying the detected associations. Furthermore, the use of a non-random sampling may have introduced selection bias and our use of self-report questionnaires may have introduced reporting bias [23]. Against this, we used validated and reliable scales to reduce measurement error.

## Conclusions

Mentalisation impairment is independently associated with increased levels of depressive symptoms. BPD symptoms appear to moderate this association which is more pronounced in the presence of greater levels of BPD symptomatology. The direction and further implications of these associations need to be clarified in a longitudinal study.

## Supplementary Information

**Additional file 1: Supplementary Table 1**. Linear regression model displaying the association between mentalisation and depressive symptoms with a fitted interaction term with borderline symptoms, adjusted for socio-demographic factors.

## Data Availability

The datasets used and/or analysed during the current study are available from the corresponding author on reasonable request.

## Abbreviations

BPD: Borderline Personality Disorder
MBT: mentalization-based treatment
MZQ: Mentalisation Questionnaire
BDI-II: Becks depression inventory -II
PAI-BOR: Personality Assessment Inventory – Borderline sub-section
MDD: Major Depressive Disorder
