# Supplementary material for: Impaired mentalizing in depression and the effects of borderline personality disorder on this relationship
Source: Borderline Personal Disord Emot Dysregul. 2021 May 4;8:15. doi: 10.1186/s40479-021-00153-x (PMC8097991; doi:10.1186/s40479-021-00153-x)
Supplement: Supplementary file 1 — Additional file 1: Supplementary Table 1. Linear regression model displaying the association between mentalisation and depressive symptoms with a fitted interaction term with borderline symptoms, adjusted for socio-demographic factors. [file 40479_2021_153_MOESM1_ESM.docx]

Supplementary Table 1 – **Linear regression model displaying the association between mentalisation and depressive symptoms with a fitted interaction term with borderline symptoms, adjusted for socio-demographic factors.**

| Regression On Depressive Symptoms | | | Coefficient (95% Ci) | P Value |
| --- | --- | --- | --- | --- |
| Mentalisation - MZQ (Per unit increase – improvement in mentalizing) | | | **0.07**  **(-0.19 to 0.34)** | **0.60** |
| Borderline Symptoms – PAI-BOR (Per unit increase – more borderline symptoms) | | | **0.86**  **(0.66 to 1.06)** | **<0.001** |
| Mentalisation#Borderline Interaction term | | | **-0.01**  **(-0.02 to -0.002)** | **0.004** |
| Age (Per Year) | | | **0.02**  **(-0.13 to 0.16)** | **0.81** |
| Years In Education (Per Year) | | | **0.19**  **(-0.26 to 0.64)** | **0.41** |
| Ethnicity | | |  |  |
|  | | White | **Baseline** |  |
|  | | Black | **0.05**  **(-4.34 to 4.43)** | **0.98** |
|  | | Mixed | **0.64**  **(-3.86 to 5.14)** | **0.78** |
|  | | Asian | **-0.01**  **(-4.42 to 4.43)** | **0.997** |
|  | | Other | **-3.59**  **(-11.08 to 3.89)** | **0.35** |
| Gender | | |  |  |
|  | | Male | **Baseline** |  |
|  | | Female | **2.73**  **(-0.40 to 5.87)** | **0.09** |
|  | | Other | **2.48**  **(-9.82 to 14.78)** | **0.69** |
| Household Income | | |  |  |
|  | | Less Than £20,000 | **Baseline** |  |
|  | | £20,000 To £35,000 | **0.60**  **(-3.08 to 4.29)** | **0.75** |
|  | | More Than £35,000 | **0.80**  **(-2.56 to 4.17)** | **0.64** |
| Employment | | |  |  |
|  | Employed | | **Baseline** |  |
|  | Student/Apprentice | | **1.68**  **(-2.05 to 5.42)** | **0.38** |
|  | Retired/Carer | | **2.55**  **(-5.30 to 10.40)** | **0.52** |
|  | Unemployed | | **4.32**  **(0.82 to 7.82)** | **0.02** |
| Constant | | | **-8.68** |  |
| Number of Observations | | | **245** |  |
| Adjusted R^2^ | | | **0.62** |  |
